# Corrigendum to “Comparison thoracic epidural and intercostal block to improve ventilation parameters and reduce pain in patients with multiple rib fractures” [J Cardiovasc Thorac Res 2011;3(3):87-91]

**DOI:** 10.5681/jcvtr.2014.017

**Published:** 2014-03-21

**Authors:** Shahryar Hashemzadeh, Khosrov Hashemzadeh, Hamzeh Hosseinzadeh, Raheleh Aligholipour Maleki, Samad EJ Golzari

**Affiliations:** ^1^Department of Thoracic Surgery, Tabriz University of Medical Sciences, Tabriz, Iran; ^2^Cardiovascular Research Center, Tabriz University of Medical Sciences, Tabriz, Iran; ^3^Medical Philosophy and History Research Center, Tabriz University of Medical Sciences, Tabriz, Iran

The authors would like to correct the name of the fifth author in this paper to Samad EJ Golzari.

